# The Role of New Inorganic Materials in Composite Membranes for Water Disinfection

**DOI:** 10.3390/membranes10050101

**Published:** 2020-05-14

**Authors:** Roberto Castro-Muñoz

**Affiliations:** Tecnologico de Monterrey, Campus Toluca. Avenida Eduardo Monroy Cárdenas 2000 San Antonio Buenavista, 50110 Toluca de Lerdo, Mexico; food.biotechnology88@gmail.com or castromr@tec.mx

**Keywords:** water disinfection, composite membranes, nanomaterials, antibacterial activity, mechanism of action

## Abstract

Today, there is an increasing interest in improving the physicochemical properties of polymeric membranes by merging the membranes with different inorganic materials. These so-called composite membranes have been implemented in different membrane-based technologies (e.g., microfiltration, ultrafiltration, nanofiltration, membrane bioreactors, among others) for water treatment and disinfection. This is because such inorganic materials (such as TiO_2_-, ZnO-, Ag-, and Cu-based nanoparticles, carbon-based materials, to mention just a few) can improve the separation performance of membranes and also some other properties, such as antifouling, mechanical, thermal, and physical and chemical stability. Moreover, such materials display specific biological activity towards viruses, bacteria, and protozoa, showing enhanced water disinfection properties. Therefore, the aim of this review is to collect the latest advances (in the last five years) in using composite membranes and new hybrid materials for water disinfection, paying particular emphasis on relevant results and new hydride composites together with their preparation protocols. Moreover, this review addresses the main mechanism of action of different conventional and novel inorganic materials toward biologically active matter.

## 1. Introduction

**Figure 1:**
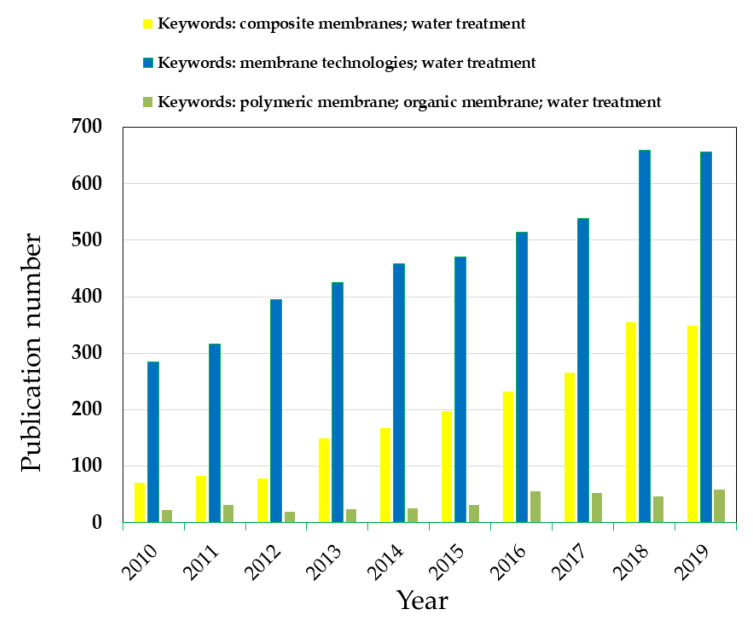
Publication record of using membrane technologies and composite membranes for water treatment over the last 10 years. Data acquired from Scopus (www.scopus.com) using the reported keywords.

Polymeric membranes have been widely applied for different types of water treatment applications, such as waste derivatives from agro-food [1,2,3], textile [4], and petroleum industries [5], or removal of organic matter from drinking water [6,7,8]. Indeed, such membranes have been implemented in several membrane-based technologies (e.g., microfiltration, ultrafiltration, nanofiltration, membrane bioreactors, among others) aiming at the water treatment and disinfection. Nowadays, the scarcity of drinking water is found among one of the main worldwide problems. Water scarcity is defined as the lack of enough water resources to meet the demands of water usage for domestic uses, drinking, irrigation, hydroelectricity production, and industrial applications [9]. Therefore, water treatment and disinfection have been one of the alternatives in satisfying such scarcity of water [10,11]. According to a World Health Organization (WHO) report, it has been documented that around 1.2 billion people in the world depend directly on treated water for consumption [12,13]. When dealing with water treatment and disinfection, the limitations of existing polymeric membranes have promoted the designing of new membrane concepts, e.g., composite membranes. A composite membrane is defined as the barrier formed by at least two structural materials (either polymer or inorganic) [14]. A composite membrane has emerged as an option of improving the physico-chemical properties of polymer membranes, such as hydrophilicity, biofouling resistance, separation performance, and mechanical, thermal, physical, and chemical stability [5]. Certainly, these membranes have been studied over recent years, gaining the attraction of researchers [15,16]. For instance, Figure 1 illustrates the trend of development works of composite membranes implemented for water treatment and disinfection, which have gained interest and application in different membrane technologies. 

In particular, when using composite membranes containing an inorganic nanomaterial (such as TiO_2_-, ZnO-, Ag-, and Cu-based nanoparticles, carbon-based materials, and graphene oxide, among others), enhanced water disinfection properties have been noticed. This is due to the fact that such inorganic materials display biological activity towards viruses [17], bacteria [18], and protozoa [19], which may allow obtaining water with high quality. Therefore, there is today an increasing interest in using inorganic-based nanomaterials in membranes for water disinfection. Therefore, the goal of this review is to release an overview of the latest development works (over the last five years) of implementing composite membranes for water disinfection, paying particular attention to the relevant insights in the field, as well as the main mechanism of action of different conventional and novel inorganic materials toward biologically active matter.

## 2. Composite Membranes for Water Disinfection: The Role of New Inorganic Materials 

**Figure 2:**
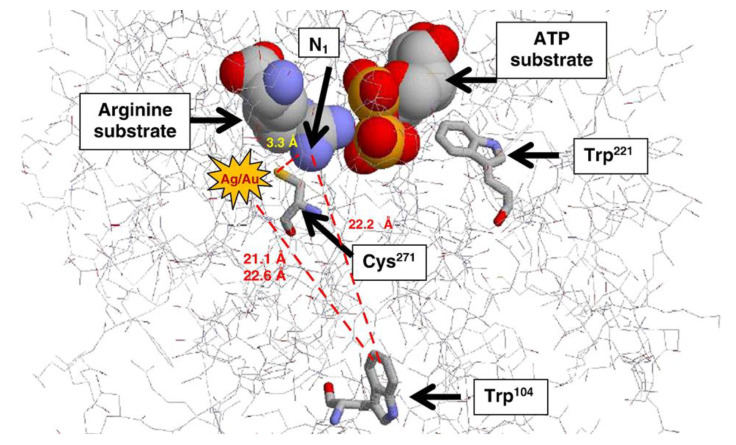
Mechanism of action for Ag/Au nanoparticles at cysteine of *Trypanosoma*. Proposed by Adeyemi and Whiteley [20].

Several examples related to the preparation of composite membranes have been reported with enhanced antibacterial properties. Such enhancements have been attributed to specific inorganic materials. This is because particular metal nanoparticles are able to mitigate the growth of bacteria and protozoan parasites. For instance, gold (Au), silver (Ag), and platinum (Pt) nanoparticles suppressed the growth of protozoa, such as *Trypanosoma b. brucei*, *T. congolense,* and *T. evansi* [19]. It has been stated that Ag and Au nanoparticles interact with arginine kinase from *Trypanosoma brucei;* the interaction occurs at a cysteine residue (as shown in Figure 2), which is crucial for the enzyme’s reaction mechanism during the enzymatic phosphoryl transfer of ADP and ATP [20]. Specifically, silver nanoparticles, like other nanoparticles such as zinc (Zn^2+^), copper (Cu^+^ or Cu^2+^), or nickel (Ni^2+^), gradually release metal ions that act antibacterially [21,22]. This action of nanoparticles is lethal to the parasites. Clearly, the selective in vitro anti-Trypanosoma action of nanoparticles has supported their promising potential as alternative anti-parasitic agents. 

**Figure 3:**
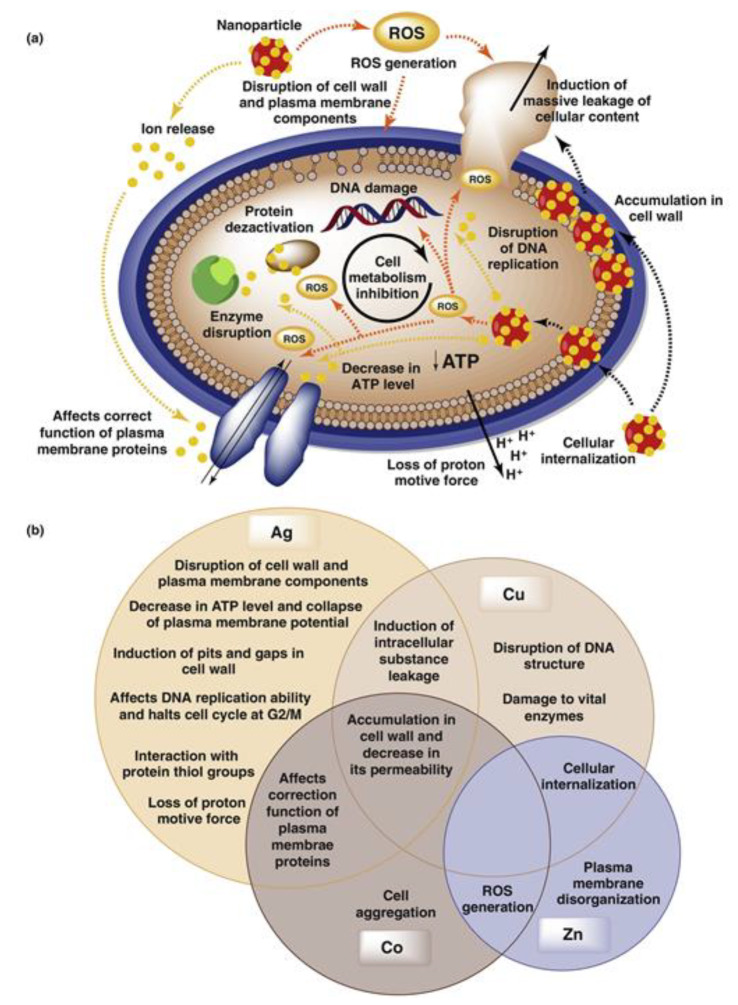
(**a**) Graphical depiction of the possible antimicrobial mechanisms of metal nanoparticles. (**b**) Effect of different metal nanoparticles (e.g., Ag, Au, Zn, Co) on the microorganisms. Taken from Wyszogrodzka et al. [21].

On the other hand, Ag-based nanoparticles have also offered antimicrobial properties. Based on such wide biological activity, several authors have incorporated these metal nanoparticles in composite membranes and then evaluated them for water disinfection applications. Several polymers, such as chitosan, cellulose acetate (CA), polyacrylonitrile (PAN), and polysulfone (PSF), have been the most popular polymeric materials used for the fabrication of Ag–nanocomposite membranes [23,24,25,26,27]. For instance, Sile-Yuksel et al. [23] evaluated the effect of Ag nanoparticle location in different polymer types, including PSF, polyethersulfone (PES), and CA. The nanoparticles were precisely identified and mapped via an EDS technique. According to the authors, the dispersion and location of the nanoparticles varied depending on the polymer, influencing the antibacterial properties of the nanocomposite membranes, e.g., the Ag nanoparticles were generally located onto the top and skin layer of the PSF and PES membranes, in which the membranes based on PSF, containing the highest nanoparticles ratio (0.09%, m/m), displayed 100% bacteriostatic efficiency towards *Escherichia coli* as a model Gram-negative bacteria. The antibacterial properties of Ag nanoparticles depend on activity several physicochemical properties of the particles, such as size, shape, and chemistry. Generally, Ag nanoparticles tend to reduce the activity of bacteria due to a synergistic effect among particle-specific biological effects, releasing Ag^+^ ions [28]. In addition, Ag nanoparticles can stick to the bacterial cell, negatively influencing the permeability and respiration of the bacteria. These particles may also affect the cell membrane producing cell lysis. In such a way, the particles can go through the bacterial cytoplasm, resulting in damage at the DNA level (see Figure 3) [29]. Regarding the separation performance, the PSF membranes increased their permeability from 200 to 235 L m^−2^ h^−1^ bar^−1^ when the Ag nanoparticles/polymer ratio increased from 0 to 0.09% [23]. As a typical phenomenon, the water permeability of membranes is generally influenced by the thickness and pore size of the skin layer and the porosity of the structure; thereby, an increase in pore size and porosity may result in an increase in water permeability [30]. Interestingly, the toxicity (or antimicrobial properties) of Ag nanoparticles depends on their physicochemical properties, e.g., it has been documented that particles smaller than 10 nm tend to be more toxic to bacteria like *E. coli* and *Pseudomonas aeruginosa* [31], while the nanoparticles ranged from 1 to 10 nm can inhibit specific viruses by binding to their gp120 glycoproteins [32]. 

Aimed at the enhancement of the physicochemical properties of Ag nanoparticles, their modification and combination with other inorganic materials were performed [33]. Li et al. [34] prepared ultrafiltration membranes modified by graphene oxide (GO) with silver nanoparticles (in the range of 0.00–0.15 wt %). These membranes were designed based on the evidence that Ag nanoparticles can be assembled on GO sheets. In theory, the GO possesses suitable functional groups (e.g., carboxyl) for the ideal nucleation sites and thus attachment of Ag nanoparticles [35]. The GO–Ag composites were embedded into polyvinylidene fluoride (PVDF) polymer; subsequently, the antibiotic effect against *E. coli* was assessed. The GO–Ag composite demonstrated a significant inhibitory effect against the bacteria together with significant enhancement in anti-bacteria adhesion and biofilm formation in the short term. The water the permeate flux was raised, from 150 to 200 L m^−2^ h^−1^, as a function of the GO-Ag loading (0.05 to 0.15 wt %) [34]. Interestingly, the authors declared that GO did not display a clear antibacterial activity (towards *Pseudomonas aeruginosa*), which is in agreement with the literature [36]. In this way, the antibacterial activity was mainly attributed to the release of Ag^+^ from the composite [34]. However, the addition of GO tends to contribute to the mechanical strength, antifouling performance, and permeation properties of the resulting membranes. For example, it has been reported that small GO loading (ca. 0.20 wt %) into PVDF ultrafiltration membranes increased the water permeability by 96.4% compared to the pristine membrane, while the tensile strength also was raised by 123% [37]. In contrast, Pramanik et al. [38] used GO and its plentiful oxygen-containing groups, and the functional groups of catechols and amines from polydopamine nanoparticles, for the removal of heavy metal decontamination. Importantly, ε-poly-L-lysine, as a natural antimicrobial peptide, was also strategically attached to the composite nanoparticles for the elimination of bacteria. Likewise, the nanocomposite had a removal efficiency over 90% towards Cr(VI), Pb(II), Hg(II), Cd(II), and Zn(II), while the same membrane fully removed (100% separation efficiency) and killed superbugs, including β-lactamase-producing *Klebsiella pneumoniae* and methicillin-resistant *Staphylococcus aureus*. The mechanism of action identified was related to mechanical wrapping, which could produce induced membrane stress, followed by disruption and damage cell membranes [39,40]. Interestingly, the efficiency data was reproduced on treating environmental river, lake, and tap water.

It is likely that the combination of Ag nano-sized particles with other materials has been the most frequent merging concept over the last 5 years, as listed in Table 1. Silver iodide/copper ferrite (AgI–CuFe_2_O_4_) hybrid material was synthesized by Zhang et al. [41]. Such complex Ag-based material showed excellent inactivation activity towards *E. coli* and *Staphylococcus aureus.* By using the material as a photocatalyst, a much higher disinfection activity was observed. This nanocatalyst was able to permeate into cells, resulting in the death of cells. According to the authors, the significant leaked potassium ion (K^+^) caused the photogeneration of a large number of reactive species, such as superoxide radical (•O_2_^−^) and holes (H^+^), which were adhered on the bacteria cell, and thus killed them. In a different approach, Shi et al. [42] also synthesized a novel Ag quantum dot (QD) /Bi_2_S_3_/SnIn_4_S_8_ Z-scheme heterostructure catalyst, which showed high inactivation activity towards *E.coli*, inactivating the bacteria after 4 h at visible light excitation. When dealing with the photoinactivation mechanism, the destruction of the cell membrane was speculated, causing the leakage and damage of intracellular biomolecules, such as DNA and protein [42].

**Figure 4:**
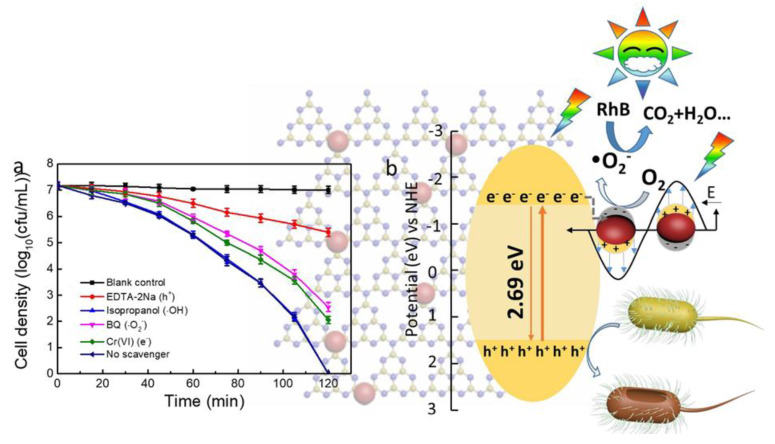
(**a**) Effect of the Ag/g-C_3_N_4_ composite on the cell density and (**b**) graphical depiction of the possible antimicrobial mechanisms and organic pollutant removal using a Ag/g-C_3_N_4_ composite. Adapted from Wei et al. [43].

Very recently, Ag-decorated graphitic carbon nitride sheets (Ag on g-C_3_N_4_) were prepared via a photodeposition method [43]. After adding the catalyst, such composites had a simultaneous antibacterial activity (towards *E. coli*) and rhodamine B (RhB) removal from the real water matrix from the Songhua River. Figure 4 (left side) shows the disinfection behavior of different samples after exposure to visible-light illumination (120 min). Since it is documented that H^+^, •OH, •O_2_^−^, and e^−^ influence the photocatalytic disinfection process, different chemical agents, such as ethylenediamine tetraacetic acid disodium salt, isopropanol, benzoquinone, Cr (VI), were proposed to trap H^+^, •OH, •O_2_^−^, and e^−^, respectively. However, it can be noticed that the highest antibacterial activity was obtained without using any of those chemical agents. Moreover, the authors also proposed the possible schematic depiction of the photocatalytic disinfection mechanism for RhB and bacteria using a 3-Ag/g-C_3_N_4_ composite (right side). When the novel material was in contact with the cells (under visible light), Ag nanoparticles released a small amount of Ag^+^ for attacking the cell membrane of the bacteria [44,45]. This study proposed by Wei and co-workers definitely provides a promising approach for developing new composites for water disinfection and pollutant degradation from real polluted water [43].

**Figure 5:**
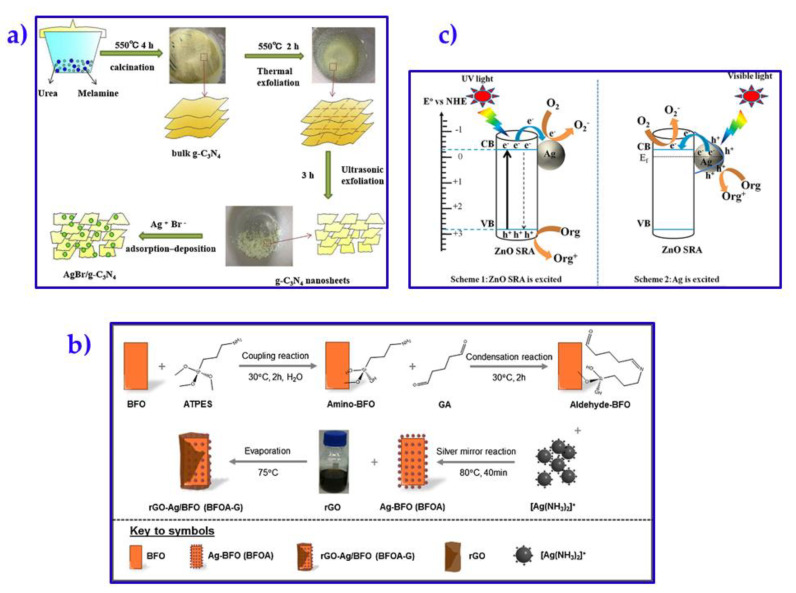
Preparation protocols of novel composite material, such as (**a**) AgBr/g–C_3_N_4_ [46], (**b**) rGO–Ag/Bi_2_Fe_4_O_9_ [48]. (**c**) Mechanism of action of Ag−ZnO in photocatalysis against organic pollutants under solar light irradiation [52].

Novel AgBr-modified g-C_3_N_4_ (AgBr/g-C_3_N_4_) photocatalysts were synthesized by Yu and co-workers [46]. In general, the new material was prepared by using a mixture of melamine and urea, followed by AgBr/g-C_3_N_4_ synthesis after thermal and sonication treatment. The preparation procedure is represented in Figure 5a. The disinfection efficiency was of about 4.80 log (during 150 min of visible light irradiation) when AgBr-modified g-C_3_N_4_ was prepared at a molar ratio of 1:5 (AgBr:g-C_3_N_4_). The modified composite presented enhanced bacterial inactivation (using *E. coli*) compared to the pristine g-C_3_N_4_ at the same experimental conditions. The improvement was associated with the effective production and transfer of the photo-induced electrons at visible light irradiation, where H^+^ played the main role for bacterial inactivation. It is important to point out that the complex material was reused for four additional cycles, showing a disinfection activity over 80% in comparison to the initial material. This can easily indicate that such a novel composite was stable and reusable for further water disinfection [46]. Wen and co-workers also synthesized Ag nanowires-carbon composites, which were embedded into a PAN/polyaniline (PANI) fibers [47]. Such fibers were effective sieve for complete removal of *E. coli* and *Staphylococcus aureus*, reaching up to 99.99% of the inactivation of the sieved bacteria. As concluding remark from authors, even if the Ag nanowire represents a challenge due to its high production cost, the composite material was potentially suggested for drinking water treatment due to its high antibacterial efficiency.

Novel nanostructured rGO-Ag/Bi_2_Fe_4_O_9_ composites have shown ultra-effective disinfection efficiencies (ca. 100%) for Gram-negative *E. coli* and *P. aeruginosa*, and Gram-positive *S. aureus* in water disinfection applications [48]. In this study, a synergistic effect was found among Bi_2_Fe_4_O_9_, Ag nanoparticles, and GO during the synthesis (see Figure 5b) and post-application in integrated technologies. Regarding the antibacterial activity towards *E. Coli*, this was noted within the first 20 min due to the Ag^+^ leaching. In addition to this, some other disinfection mechanisms occur, such as GO-assisted Ag^+^ release, Ag-assisted Fenton reaction, Ag/rGO co-assisted photocatalysis, and Ag-assisted photo-Fenton oxidation [49,50]. All these involved mechanisms lead to the membrane cell damage produced by reactive oxygen species.

Jiang et al. [51] performed the in-situ photocatalytic synthesis of Ag nanoparticles by crumpled GO membranes for filtration and disinfection. These nanocomposite membranes exhibited around 3 log inactivation for both *Escherichia coli* and *Bacillus subtilis*, demonstrating an improved antimicrobial activity that other composite membranes (e.g., GO−TiO_2_ nanocomposites). Regarding the water permeation, such Ag–GO composite membranes showed a water flux of about 250 L m^−2^ h^−1^ bar^−1^, together with the acceptable rejection of methyl orange (ca. 24%) and bovine serum albumin (ca. 64%). It is important to point out that TiO_2_ composite materials are also offering interesting disinfection performance, e.g., Liu et al. [53] generated a TiO_2_-based photocatalyst (i.e., P/Ag/Ag_2_O/Ag_3_PO_4_/TiO_2_) to analyzed its bactericidal activity. In practice, after photocatalytic evaluation varying different operating parameters (e.g., light wavelength, light intensity, temperature, solution pH, and inorganic ions), the composite inactivated up to 10^8^ CFU mL^−^^1^ of *E. coli* within the first 40 min (using only 0.5 g L^−^^1^ catalyst loading). By exploring the cell morphology of the bacteria, the researchers also found strong morphology changes of *E. coli* cells, which was proved by determining K^+^ leakage in cells. The inactivation was mainly attributed to the production of reactive species (such as H^+^ and •O_2_^−^) together with the leakage of Ag^+^.

The merging of silver with other metal nanoparticles, like zinc oxide (ZnO), has also been recently studied [52]. Nano-sized ZnO has been widely used in different applications, including coatings, paints, sunscreens, among others [54,55]. Within their main properties, the nanoparticles have demonstrated notably antibacterial activity on different types of bacteria, such as *Staphylococcus aureus* [56,57], *Escherichia coli* [56], *Bacillus subtilis* [58], and *Streptococcus agalactiae* [59]. Based on these attributes, Liu et al. [52] manufactured Ag−ZnO submicrometer rod arrays into polycarbonate membranes via electrochemical deposition. Such composite membranes have acceptable antibacterial properties against *E. Coli*. According to the authors’ findings, the Ag addition into ZnO also improved the photocatalytic degradation of Congo Red (up to 91.9%) since it suppressed the recombination of e^-^ and H^+^. In principle, ZnO has demonstrated antibacterial capacity; however, its mechanism of action was not clear. Different mechanisms have been suggested, such as the photocatalytic generation of hydrogen peroxide, cell membrane disruption (see Figure 5c), as well as the Zn^2+^ ion binding to the cell membrane that can delay the lag phase within the microbial growth [60]. 

Interestingly, Ag nanoparticles have demonstrated the ability to inactivating several pathogenic viruses, such as human immunodeficiency virus 1, hepatitis B virus, and influenza A [61,62,63]. Likewise, Ag-based composites inactivated influenza A. This was demonstrated by Park et al. [64], who synthesized Ag nanoparticle-decorated silica hybrid composites. The particles of ~400 nm in diameter exhibited remarkable anti-influenza A effects in a dose-dependent manner (after 1 h of exposure). In addition to this, due to their nonspecific interactions with biomolecules, the hybrid particles may inactivate several influenza strains without significant resistance. 

With the aim of producing drinking water, Arivizhivendhan et al. [12] developed a bioactive prodiprodigiosin-conjugated iron-oxide-activated carbon composite ((Ac)F@Fe_3_O_4_−PG) for the efficient removal of biological contaminants, especially for the disinfection towards *E. coli* and *Bacillus subtilis.* Indeed, the composite provoked a cell membrane damage related to the surface charge neutralization by cationic (Ac)F@Fe_3_O_4_−PG, which affected the transport systems of the bacteria. Meanwhile, the production of reactive oxygen substances also contributed to cell death due to changes similar to apoptosis in cellular morphology. Finally, the authors concluded that the Fe-based composite material also demonstrated reusability, and it displayed good long-term antibacterial activity. Considering this study, iron oxide nanoparticles have acquired the interest of chemists in solving environmental issues due to their versatility, separation performance, biocompatibility, and substantial surface groups [65,66], which play an important role in disinfection applications. Fe_2_O_3_ nanoparticles were filled into polyacrylonitrile (PAN) membranes [67]. The resulting nanocomposite Fe_2_O_3_@PAN membrane (containing with 52.7 wt % nanoparticle loading) were evaluated for the treating of phosphate solutions, showing a flux in the order of 670–800 L m^−2^ of sterile water with less than 20 μg/L phosphorus. Also, this membrane inhibited microbial regrowth in the treated water [67].

**Figure 6:**
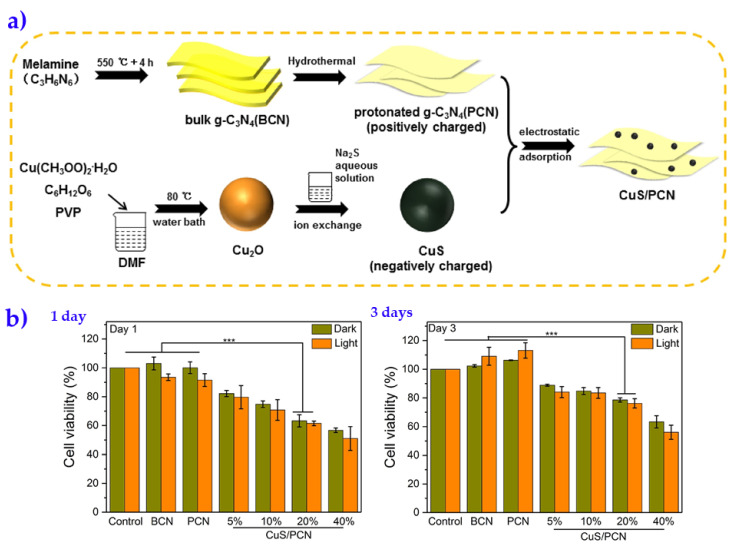
(**a**) Synthesis of CuS/protonated g-C_3_N_4_ composites, and (**b**) their effect on the cell viability as a function of the composite loading. Adapted from Ding et al. [71].

Different metal-based nanocomposite materials have also been developed for water disinfection. For instance, mixed matrix cross-linked PVA–Cu nanocomposite fibers have shown over 99.5% filtration efficiency in removing *E. coli* [68]. Certainly, copper is well known for its strong antibacterial activity; for example, it is recognized to kill 99.9% of pathogens in 2 h. Commonly, copper ions can kill bacteria on direct contact due to DNA degradation [69,70]. Based on such evidence, the modification or hybridization of Cu nanomaterials has been a current trend in the manufacture of new effective composite materials. Ding et al. [71] synthesized CuS/protonated g-C_3_N_4_ composites; the synthesis procedure is provided in Figure 6a. It is important noting that the authors proposed the synthesis of this hybrid material due to the documented photodynamic and photothermal activities of the pristine materials CuS nanoparticles and g-C_3_N_4_, respectively. Likewise, when such composite material contains 20% CuS, the composite clearly displays a synergistic effect for both photothermal and photocatalysis under light irradiation in a short time (~20 min). The corresponding bacteria-killing efficiencies against *Staphylococcus aureus* and *Escherichia coli* were 98.23% and 99.16%, respectively. Regardless of dark or light, an increase of CuS content in composites resulted in fewer live cells (see Figure 6b), which could be attributed to the native toxicity of the released Cu^2+^ [72]. Interestingly, after three days of culture and cell metabolism, the cell viability of BCN and PCN was greater than that of the control at the absence and presence of light, providing an idea of the excellent biosafety of the material. Based on their findings, the authors declared that this new composite represents a promising material for different environmental disinfection, including water and some other sterilization tasks. Today, several researchers are attracted by g-C_3_N_4_ since it exhibits meaningful antibacterial activity together with low cost and chemical stability [73,74].

Recently, many nano-sized surface-modified iron oxide structures have been developed for the disinfection of pathogens in the aqueous solution. For example, Tong et al. [75] tailored Fe_3_O_4_-deposited flower-like MoS_2_ nanocomposites for disinfection (against *E. coli*) and pharmaceutical degradation (such as diclofenac). Especially, the mechanism of action of this nanocomposite implied a Fenton reaction, in which the disinfection against the bacteria and degradation of diclofenac was due to the generation of •OH by reacting Fe(II) and H_2_O_2_. At catalytic conditions, the composite efficiently inactivated *E. coli*, and remove diclofenac at a wide pH range from 3.5 to 9.5. In addition to this, 1.2 × 10^6^ CFU mL^−1^ cells were fully disinfected by the Fe_3_O_4_-deposited flower-like MoS_2_ nanocomposites within 30 min at pH 6 containing 5 mM H_2_O_2_. On the other hand, around 10 mg L^−1^ DCF was totally degraded in 7 min using 1 mM H_2_O_2_ (at pH 6). Moreover, the total organic carbon (TOC) removal in municipal wastewater increased by the action of sonication protocol, and more efficiently at longer exposure times (between 3–4 h) [75]. Concurrently, the development of Fe^0^/Fe_3_O_4_ composites for phosphate removal from river water was recently performed by Wang et al. [76]. The interest in removing phosphorus deals with eutrophication. In general, the excess phosphorus in natural waters facilitates the proliferation of certain algae. Therefore, there is a need to preserve a low concentration of phosphorous in natural waters and thus have an environmental equilibrium. Wan and co-workers [76] reduced the phosphate from 0.4 to 0.010 mg-P L^−1^ by using only 0.5 g L^−1^ of Fe^0^/Fe_3_O_4_ coming from 0.5 mg L^−1^ Fe^2+^. Herein, it was likely that the resulting treated river water met the WHO drinking water standards after 80 min of reaction. The high phosphate removal was properly a contribution of adsorption, co-precipitation, and precipitation. During the adsorption identified as the primary removal mechanism, it involved attraction and inner-sphere complexation.

A high-performance water purification composite membrane was prepared by Manohara et al. [77], who designed a highly oxygenated and aluminum-functionalized solvothermal carbon (Al–STC) composite. Practically, such a carbon-based membrane has tremendous performance in terms of pollutant removal. Briefly, the membrane removed about 99.9% malachite green with a flux of 1522 L m^−2^ h^−1^, while the removal of methylene blue was >99.9% and flux 885 L m^−2^ h^−1^. When removing pharmaceutical derivatives, it also displayed excellent separation performance, such as ciprofloxacin (rejection >99.9%, flux = 1011 L m^−2^ h^−1^), paracetamol (rejection 53%, flux = 1010 L m^−2^ h^−1^), oxytocin hormone (rejection 88.6%, flux = 955 L m^−2^ h^−1^), surfactant CTAB (rejection 94.9%, flux = 1436 L m^−2^ h^−1^), and heavy metal with Cr(VI) (rejection over 99.9% and flux 932 L m^−2^ h^−1^). Unfortunately, such membranes were not tested for their antibacterial capacity. Similarly, Wang and co-workers [78] did not evaluate the antibacterial capacity of a novel ZIF-8 with carbon dot (CDs@ZIF-8) modified thin-film polyamide nanocomposite membrane; however, such membranes in a reverse osmosis process exhibited a rejection removal of about 95% towards disinfection byproducts. Thanks to the large surface area and plenty of oxygen-containing groups of CDs@ZIF-8, the hybrid nanoparticles acted as a nanocarbon filler, with a high adsorption capacity of disinfection byproducts such as trihalomethanes, haloacetonitriles, and haloketones. After chlorination, the percentage reduction for salt rejection rate (separating a NaCl solution containing 2000 ppm) of the CDs@ZIF-8 thin-film composite membranes was lower than that of the unfilled thin-film membranes due to hydrogen bonding between the carbon dots and the polymer (i.e., polyamide), replacing amidic hydrogen with chlorine, making the membrane less susceptible to chlorine attack and thus enhancing chlorine-resistance. Regarding the water permeability of such membranes, such property varied as a function of the hybrid nanoparticle loading; it increased from 1.1 L m^−2^ h^−1^ bar^−1^ in unfilled thin membrane up to 1.9 L m^−2^ h^−1^ bar^−1^ in the composite membranes containing 0.15 wt % loading of CDs@ZIF-8 [78]. Carbon-based nanomaterials are currently used in membranes to obtain superior performance [15,79,80]; one of these promising materials has been carbon nanotubes (CNTs). CNTs have inactivated *E. coli* K12 by means of ohmic heating. Nevertheless, according to Oh’s hypothesis, there is still a research gap in understanding the effect of surface modification of hydrophobic CNT interfaces and electrical heating conditions [81]. Thereby, Oh and co-workers studied the effect of CNT-loading into polycarbonate membranes by applying the layer-by-layer technique. Before that stage, an oxygen plasma protocol was applied to the composite membranes for the functionalization of the CNTs, followed by attaching copper tapes onto the CNTs layer, which served as electrodes for the ohmic heating. Such composites membranes were then tested for the removal of *Legionella pneumophila* suspension (ca. 20,000 CFU mL^−1^). Indeed, there was an effective removal of 99.99% of the cells, noting a cell-free permeate. The membranes also displayed a high permeation flux in the range of 1502–1600 L m^−2^ h^−1^ bar^−1^ [81].

**Figure 7:**
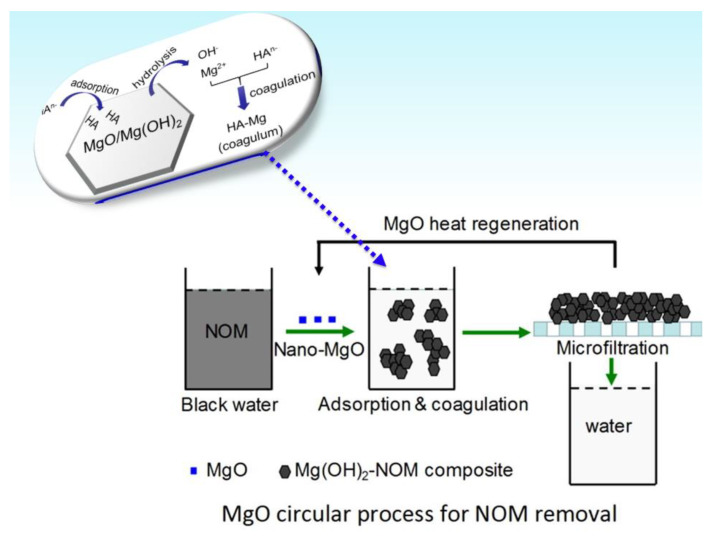
Representation of the natural organic matter removal approach by recycling MgO. Adapted from Zhou et al. [84].

Magnesium oxide (MgO) is an effective and cheap inorganic material that displays relevant adsorption capacity [82]. MgO has even demonstrated its ability for the adsorption of selenium ions [83]. Based on the evidence that MgO can hydrolyze and form Mg(OH)_2_ and thus provide authigenic Mg^2+^ in the presence of water and turn back to MgO, Zhou et al. [84] proposed the use of MgO as a coagulant aimed at the removal of natural organic matter from water and subsequently recycling MgO by heat regeneration, as represented in Figure 7. 

Nano-sized MgO was dispersed in a solution containing humic acid (ca. 1.0 g of sodium humate in 0.5 L water) as the most representative natural organic matter; afterward, the suspension was filtered using a commercial microfiltration membrane (PES, 0.45 µm; see Figure 7). According to Zhou’s findings [84], the proposed approach presented several positive insights, e.g., the MgO displayed relevant natural matter removal capacity, while the dissolved Mg^2+^ was able to remove about 92% of the natural organic matter through the coagulation. Moreover, the generated Mg(OH)_2_ was found as the main responsible for the adsorption mechanism of the humic acid, and finally, the authors declared that the nano-sized MgO can be recycled more than 10 times without the production of any waste material. This contribution definitely represents a valuable approach in terms of sustainable production of drinking water purification and some other field, such as wastewater remediation and treatment [84]. 

To date, emerging and new hybrid composite nanoparticles are commonly merged with chemically synthesized polymers. Today, there is a clear trend of using biopolymers, aiming for the replacement of those synthetic polymers. Different biopolymers (e.g., chitosan, sodium alginate, polylactic acid, among others) have been more deeply studied for membrane separation techniques, such as MF, UF, NF, gas separation, and pervaporation [85,86,87]. Unfortunately, few studies on using biopolymers in composite materials for water disinfection have been developed. One of the current development works in the field is the one reported by Majiya et al. [88], who modified chitosan membranes using pyromellitic dianhydride. Crucially, such modification was aimed at the incorporation of carboxyl groups and facilitation of electrostatic adsorption of the highly basic photosensitizers like 5-, 10-, 15-, and 20-tetrakis (1-methyl-4-pyridinio) porphyrin tetra p-toluene sulfonates (TMPyPs). The composite membrane (chitosan–TMPyP) was subsequently evaluated for the photodynamic inactivation of bacteriophage (e.g., MS2) and *E. coli*. For example, the bacteriophage number was decreased from 8 to 0 log PFU mL^−1^ within the first 50 min of illumination exposure. The efficiency of such composite material was not reduced when reused at least 3 times. At this point, the material and its reusability represent a good candidate to reduce costs within water purification and disinfection applications.

## 3. Concluding Remarks and Suggestions for the New Readers in the Field

Through the analysis of the latest research works, composite materials and membranes have demonstrated their ability to degrade several pollutant compounds and microorganisms contained in water. Compared to the last decades, relevant efforts of synthesizing new emerging hydride composite materials based on TiO_2_-, ZnO-, Ag-, Fe-, Mg-, and Cu-based nanoparticles, and carbon-based material, have been performed. These new materials, reported in the current review, have released interesting insights in the field of removing bacteria, viruses, and bacteriophage, with high removal efficiency (over 99%). Moreover, the current review addresses the main preparation protocols used for the fabrication of such hybrid composites, and importantly, the primary mechanism of action of such developed materials is stated. Finally, according to the review focused on the studies of the last five years, the following suggestions/recommendations to new scientists in the field are given:

- It is needed to develop low-cost composite materials and membranes. In general, the membranes, as well as the primary reactive materials of the composites, represent the main cost of the overall disinfection processes.
- Commonly, the synthesis procedures and techniques for new hybrid composites are generally implemented at a lab-scale. Based on the excellent antibacterial performance, researchers should start to implement and develop protocols for the preparation of large-scale composites and membranes.
- To date, most of the studies have evaluated the disinfection activity of composite membranes and composite materials using model water solutions; it is recommended to start the testing of hybrid composites using real complex aqueous solutions since few reports have used natural complex water systems (e.g., natural water coming from rivers, lakes, and taps) [38,43,76].
- It is recommended to evaluate the reusability of new hybrid composites after testing their antibacterial activity and pollutant removal. There are few studies addressing the evaluation of the reuse of such materials [12,88]. In this way, the overall disinfection cost could be reduced. Moreover, there is also a big need for developing long-term operation tests to analyze the stability of such new materials.
- The new researchers in the field should also initiate the exploration of other biopolymers in the preparation of composite membranes. Today, there is a trend of using more environmentally friendly materials in accordance with the current environmental regulations.
- Finally, the mechanism of action of the different composite materials has been given. At this point, new researchers aiming for the development of new composites should provide a good understanding of the action of the developed hybrid materials.

## Figures and Tables

**Table 1:** Recent studies on combining Ag nanoparticles with other materials.

Ag Composite	Polymer Matrix	Antibacterial Activity	Performance	Ref.
GO–Ag	PVDF	*Escherichia coli*	Flux: 150–200 L m^−2^ h^−1^	[34]
GO–Ag	*-*	*Pseudomonas aeruginosa*	Efficiency *: 100%	[36]
AgI–CuFe_2_O_4_	*-*	*Staphylococcus aureus* *Escherichia coli*	-	[41]
Ag QDs decorated Z-scheme Bi_2_S_3_/SnIn_4_S_8_	*-*	*Escherichia coli*	Efficiency *: 84%	[42]
Ag/g–C_3_N_4_	*-*	*Escherichia coli*	Efficiency *: 99%	[43]
AgBr/g–C_3_N_4_		*Escherichia coli*	Efficiency *: 80%	[46]
PAN/PANI/AgNWs–CC	PAN	*Escherichia coli* *Staphylococcus aureus*	Efficiency *: 100%	[47]
rGO–Ag/Bi_2_Fe_4_O_9_	*-*	*Escherichia coli* *Staphylococcus aureus Pseudomonas aeruginosa*	Efficiency *: 100%	[48]
Ag-crumpled GO nanocomposite	PES	*Escherichia coli* *Bacillus subtilis*	Flux: 454 L m^−2^ h^−1^	[51]
Ag–ZnO	*-*	*Escherichia coli*	Efficiency *: 92%	[52]
P/Ag/Ag_2_O/Ag_3_PO_4_/TiO_2_	*-*	*Escherichia coli*	Efficiency *: 100%	[53]

* Disinfection efficiency.
